# MRI features of and factors related to ankle injuries in asymptomatic amateur marathon runners

**DOI:** 10.1007/s00256-020-03530-9

**Published:** 2020-07-06

**Authors:** Wanzhen Yao, Yanjing Zhang, Li Zhang, Jing Zhou, Yi Zhang, Xiaozhong Zheng, Jianping Ding

**Affiliations:** 1 Department of Radiology, The Affiliated Hospital of Hangzhou Normal University, No. 126, Wenzhou Road, Gongshu District, Hangzhou, 310000 Zhejiang China; 2 Department of Radiology, The Children’s Hospital, Zhejiang University School of Medicine, National Clinical Research Center for Child Health, Hangzhou, Zhejiang China

**Keywords:** Marathon, Ankle joint, Injuries, Magnetic resonance imaging

## Abstract

**Objective:**

To analyze the MRI manifestations of and factors related to ankle injuries in asymptomatic amateur marathon runners.

**Materials and methods:**

A total of 113 amateur marathon runners without any ankle joint symptoms were recruited. Each participant was asked to complete a questionnaire at the beginning of the study and underwent MRI of the ankle. The MRI manifestations of ankle injuries were summarized, and binary logistic regression analysis was applied to analyze the factors related to ankle injuries.

**Results:**

The main MRI features were bone marrow edema-like signal intensity, peritendinous effusion, and partial lateral collateral ligament injury. Others included Achilles tendinopathy, cyst-like lesions, osteochondral lesions, and subcutaneous soft tissue edema. The risk factor for bone marrow edema-like signal intensity in amateur marathon runners was a rearfoot strike pattern (*p* = 0.028, OR = 1.172); the risk factors for peritendinous effusion were a higher weekly running distance (*p* = 0.013, OR = 1.685) and increased running years (*p* = 0.039, OR = 1.113), whereas a rearfoot strike pattern (*p* = 0.005, OR = 0.831) was a protective factor for peritendinous effusion; the risk factor for Achilles tendinopathy was increased age (*p* = 0.008, OR = 1.412); the risk factors for anterior talofibular ligament injury were a rearfoot strike pattern (*p* = 0.017, OR = 1.346) and higher weekly running distance (*p* = 0.022, OR = 1.171); and the factors for calcaneofibular ligament injury were a higher weekly running distance (*p* = 0.029, OR = 1.570) and rearfoot strike pattern (*p* = 0.035, OR = 1.463).

**Conclusion:**

The main MRI features of asymptomatic amateur marathon runners are bone marrow edema-like signal intensity, peritendinous effusion, and partial lateral collateral ligament injury. In addition, increased age, increased running years, higher weekly running distance, and different foot strike patterns are risk factors for ankle injuries.

## Introduction

Marathon running is a popular pastime and sport activity, with a total of 7,125,600 participants reported in China in 2019. Marathon running appears to have physical and psychological benefits. Unfortunately, the increased popularity of marathon running is related to a rise in the number of running-related injuries (RRIs). The RRI incidence rate reportedly ranges from 2.5 to 33.0 injuries per 1000 h of running [1]. Injuries to the lower limb are common among RRIs, with knee (28%), ankle-foot (26%), and shank (16%) injuries being the highest [2]. The ankle joint is the main load-bearing joint of the lower limb during marathon running. Due to various internal and external factors, injuries to bone, cartilage, tendons, and ligaments are prone to occur, and magnetic resonance imaging (MRI) is the preferred imaging method for evaluating these injuries. It is of particular concern whether excessive repetitive musculoskeletal stress, especially in marathon running, causes lesions of the ankle. A previous study suggested the presence of bone marrow edema-like signal intensity in the ankle and foot of marathon runners after a race [3]. This phenomenon is probably due to the increased stress from repetitive external impact loading during running. Another MRI study showed several abnormalities such as bone marrow edema-like signal intensity, signal alteration within the soleus muscle, small punctate hyperintensities within the Achilles tendon, and an increased amount of fluid in the retrocalcaneal bursa in the ankle and foot both after marathon races and in asymptomatic physically active individuals without any preceding extraordinary strain [4]. There are only a few studies about the MRI characteristics of the ankle and the factors related to injuries in amateur marathon runners. Thus, the questions of whether marathon running causes lesions of the ankle and to what extent marathon running causes lesions of the ankle have not been fully answered. The aims of this study were to investigate whether marathon running causes lesions in bone, cartilage, tendons, ligaments, and/or the subcutaneous soft tissue of the ankle using MRI and to analyze the factors related to ankle injuries.

## Materials and methods

### Participants

A total of 113 amateur marathon runners (63 males, 50 females) were recruited from the Zhejiang University Entrepreneurs Outdoor Association. Amateur marathon runners were defined as those who had not participated in formal training and whose occupation was not marathon running. A questionnaire was used to record the basic information of the participants, including name, sex, age, occupation, height, weight, running years, running pace, weekly running distance, foot strike pattern, running experience, number of marathons, and previous injuries. All participants were asked to sign an agreement to ensure the authenticity of the questionnaire content to the maximum extent. The characteristics of the study participants are shown in Table 1. The main inclusion criteria were as follows: running for more than 1 year, running more than 3 times per week, each running distance was not less than 10 km, participating at least once in a formal half-marathon in the past year, having no ankle pain and discomfort or present and previous ankle injuries, and no contraindications for undergoing MRI. The exclusion criteria included the following: professional marathon runners, presence of known ankle problems, previous ankle surgeries, or poor cardiovascular health. The study received ethical approval by the Affiliated Hospital of Hangzhou Normal University Research Ethics Committee and informed consent was obtained from all participants.

**Table 1:** Baseline characteristics of subjects (*n* = 113)

Characteristics	
Age (years)	43.1 ± 6.5 (26.0–58.0)
BMI (kg/m^2^)	22.6 ± 2.4 (16.4–26.7)
Running years (years)	4.8 ± 3.1 (1.0–12.0)
Running pace (min/s/km)	5: 40 ± 0: 15 (5: 00–7: 00)
< 6:00 (faster running pace)	62 (54.9)
≥ 6:00 (slower running pace)	51 (45.1)
The weekly running distance (km)	56.3 ± 11.5 (40.0–80.0)
< 60 (lower weekly running distance)	70 (61.9)
≥ 60 (higher weekly running distance)	43 (38.1)
Sex	
Male	63 (55.8)
Female	50 (44.2)
Foot strike pattern	
Non-rearfoot strike pattern	53 (46.9)
Rearfoot strike pattern	60 (53.1)

Data are presented as mean ± SD (range) or *n* (%)

*BMI* body mass index, *min/s/km* minute/second/ kilometer

### Magnetic resonance imaging

The 113 participants did not perform any form of exercise for 3 days before the MRI examination to avoid the short-term effects of exercise on the ankle. All participants were scanned with the same 1.5 T MRI scanner (Magnetom Avanto, Siemens Healthcare, Germany) with a dedicated 8-channel ankle coil. The imaging protocols included a *T*_1_-weighted imaging (T_1_WI) sequence in sagittal plane [repetition time (TR), 520 ms; echo time (TE), 10 ms; section thickness, 3 mm; intersection gap, 0.3 mm; field of view (FOV), 16 × 16 cm], a fat-suppressed protein density-weighted imaging (fs-PDWI) sequence in the sagittal (TR, 2600 ms; TE, 45 ms; section thickness, 3 mm; intersection gap, 0.3 mm; FOV, 16 × 16 cm) and coronal planes (TR, 2700 ms; TE, 45 ms; section thickness, 3 mm; intersection gap, 0.3 mm; FOV, 16 × 16 cm) and a fat-suppressed T_2_-weighted imaging (fs-T_2_WI) sequence in axial plane (TR, 4000 ms; TE, 94 ms; section thickness, 3 mm; intersection gap, 0.3 mm; FOV, 16 × 16 cm). The total acquisition time per ankle scan was 12 min. All participants were asked to complete the MRI safety screening form, informed consent form, and questionnaire before undergoing MRI.

### Imaging analyses

Ankle MRI observations included bone, cartilage, tendons, ligaments, and the surrounding subcutaneous soft tissue. Bone injuries included bone marrow edema-like signal intensity, cyst-like lesions, and stress fractures in the distal tibia, distal fibula, talus, calcaneus, and the navicular, medial, middle, and lateral cuneiform, and cuboid bones. The integrity and signal features of the tendons and the presence of fluid in the tendon sheath were determined. The following tendons and the tendon sheaths were evaluated: tibialis posterior, flexor hallucis longus, flexor digitorum longus, peroneal, tibialis anterior, extensor hallucis longus, and extensor digitorum longus. Ligament injuries included partial tears and complete tears. The following ligaments were evaluated: anterior talofibular ligament, posterior talofibular ligament, calcaneofibular ligament, and deltoid ligament. Osteochondral lesions were mainly observed in the talar dome. The soft tissue was mainly observed for edema.

Two specialized musculoskeletal radiologists diagnosed the MRI images independently. In case of discrepancies between the radiologists’ reports concerning the findings, the final diagnosis result was determined by the third senior professional musculoskeletal radiologist.

### Statistical analyses

Statistical analysis was performed with SPSS Version 19.0 software for Windows. We compared the inter-observer agreement on the MRI readings with kappa coefficient analysis, which takes into account the degree of disagreement between radiologists. Descriptive statistics were used to describe the baseline characteristics of all participants using mean values and standard deviations (SDs) or numbers and percentages (%). Since 43 participants (26 males, 17 females) only had their right ankle examined for personal reasons (e.g., they could not undergo bilateral ankle scans for too long), the analysis of factors related to ankle injuries was based on the right ankle MRI findings for all participants. Multivariable analyses were performed with a binary logistic regression model in which each variable with a *p* value of < 0.05 was entered into the model. The results of the logistic regression analysis were expressed as odds ratios (ORs) with corresponding 95% confidence intervals [95% confidence intervals (CIs)].

## Results

A total of 183 ankles were included from 113 asymptomatic amateur marathon runners, 100 ankles were from 63 male participants, and 83 ankles from 50 female participants. Bone marrow edema-like signal intensity [3], peritendinous effusion [4], and ligament injuries [5] were graded according to prior studies, and a grade scale was used (Table 2). The MRI results and inter-observer kappa values are shown in Table 3.

**Table 2:** Grading system used in the evaluation of MRI examinations

MRI features	Grade 0	Grade 1	Grade 2	Grade 3
Bone marrow edema-like signal intensity [3]^a^	Normal signal	Increased signal intensity involving< 25% of the bone	Increased signal intensity involving 25–50% of the bone	Increased signal intensity involving> 50% of the bone
Peritendinous effusion [4]^a^	Radius of effusion collection less than 0.25 times the diameter of tendon	Radius of effusion collection more than 0.25 times and less than 1.0 times the diameter of tendon	Radius of effusion collection greater than 1.0 times the diameter of tendon	–
Ligament injuries [5]^a^	Normal signal	Intact ligament and surrounding fascial edema	Partial tear, with incomplete disruption of the fibers and surrounding edema	Complete tear with more extensive edema

^a^Reference number for scaling

**Table 3:** Summary of MRI results (*n* = ankles)

	Total injuries		Grade 0		Grade 1		Grade 2		Grade 3		Inter-observer kappa
	*n*	%	*n*	%	*n*	%	*n*	%	*n*	%	
Bone marrow edemalike signal intensity											
Distal tibia	12	6.6	171	93.4	4	2.2	6	3.3	2	1.1	0.75
Distal fibula	2	1.1	181	98.9	2	1.1	0	0	0	0	0.88
Talus	29	15.8	154	84.2	9	4.9	14	7.6	6	3.3	0.69
Calcaneus	35	19.1	148	80.9	15	8.2	12	6.5	8	4.4	0.71
Navicular	6	3.3	177	96.7	1	0.5	1	0.5	4	2.2	0.85
Ectocuneiform	3	1.6	180	98.4	1	0.5	0	0	2	1.1	0.85
Peritendinous effusion											
Posterior tibialis tendon	145	79.2	16	8.7	97	53.0	32	17.5	–	–	0.57
Flexor digitorum longus tendon	78	42.6	13	7.1	44	24.0	21	11.5	–	–	0.59
Flexor hallucis longus tendon	131	71.6	12	6.6	63	34.4	56	30.6	–	–	0.53
Peroneus longus and brevis tendon	32	17.5	22	12.0	10	5.5	0	0	–	–	0.62
Anterior tibialis tendon	19	10.4	15	8.2	4	2.2	0	0	–	–	0.70
Extensor hallucis longus tendon	10	5.5	8	4.4	2	1.1	0	0	–	–	0.68
Extensor digitorum longus tendon	17	9.3	15	8.2	2	1.1	0	0	–	–	0.71
Ligament injuries											
Anterior talofibular ligament	87	47.5	96	52.5	57	31.1	30	16.4	0	0	0.59
Posterior talofibular ligament	46	25.1	137	74.9	31	16.9	15	8.2	0	0	0.68
Calcaneofibular ligament	72	39.3	111	60.7	45	24.6	27	14.7	0	0	0.56
Deltoid ligament	28	15.3	155	84.7	16	8.7	12	6.6	0	0	0.61
Achilles tendinopathy	49	26.8	–	–	–	–	–	–	–	–	0.51
Achilles tendon effusion	21	11.5	–	–	–	–	–	–	–	–	0.56
Cyst-like lesions	10	5.46	–	–	–	–	–	–	–	–	0.84
Talus	7	3.82	–	–	–	–	–	–	–	–	
Calcaneus	3	1.64	–	–	–	–	–	–	–	–	
Osteochondral lesions	6	3.3	–	–	–	–	–	–	–	–	0.60
Subcutaneous soft tissue edema	25	13.7	–	–	–	–	–	–	–	–	0.63

**Fig. 1:**
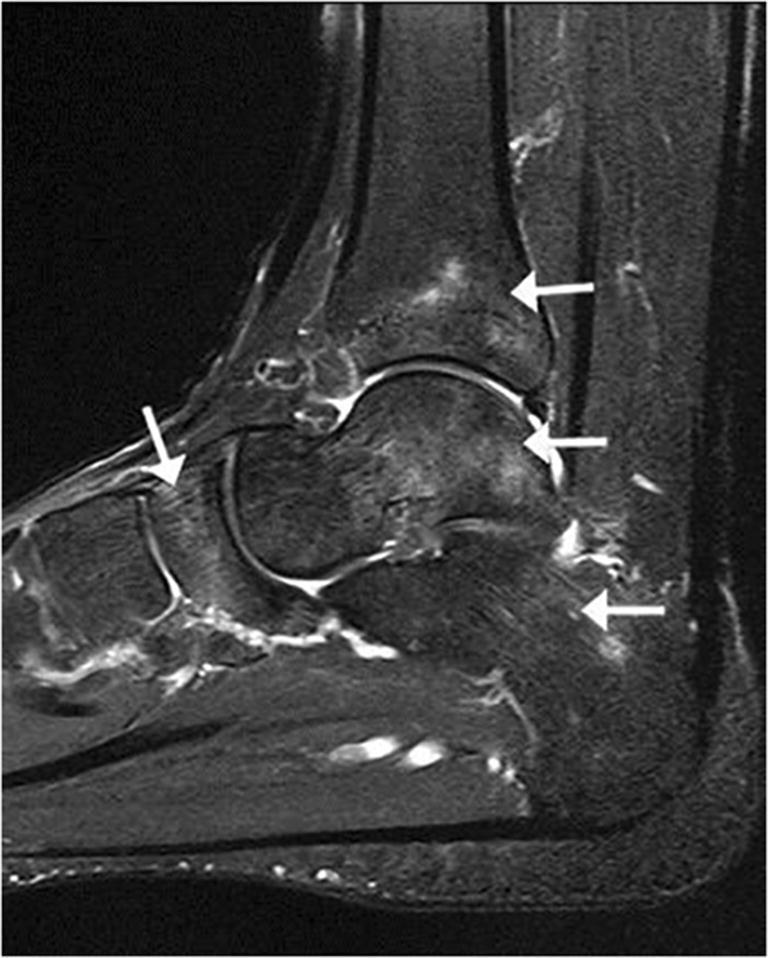
A 36-year-old male amateur marathon runner with 2 years of running experience, a running pace of 5:30 min/s/km, a weekly running distance of 65 km, and a rearfoot strike pattern. Sagittal fat-suppressed protein density-weighted imaging (fs-PDWI) showing grade 3 bone marrow edema-like signal intensity of the distal tibia, talus, calcaneus, and navicular bone (arrow)

**Fig. 2:**
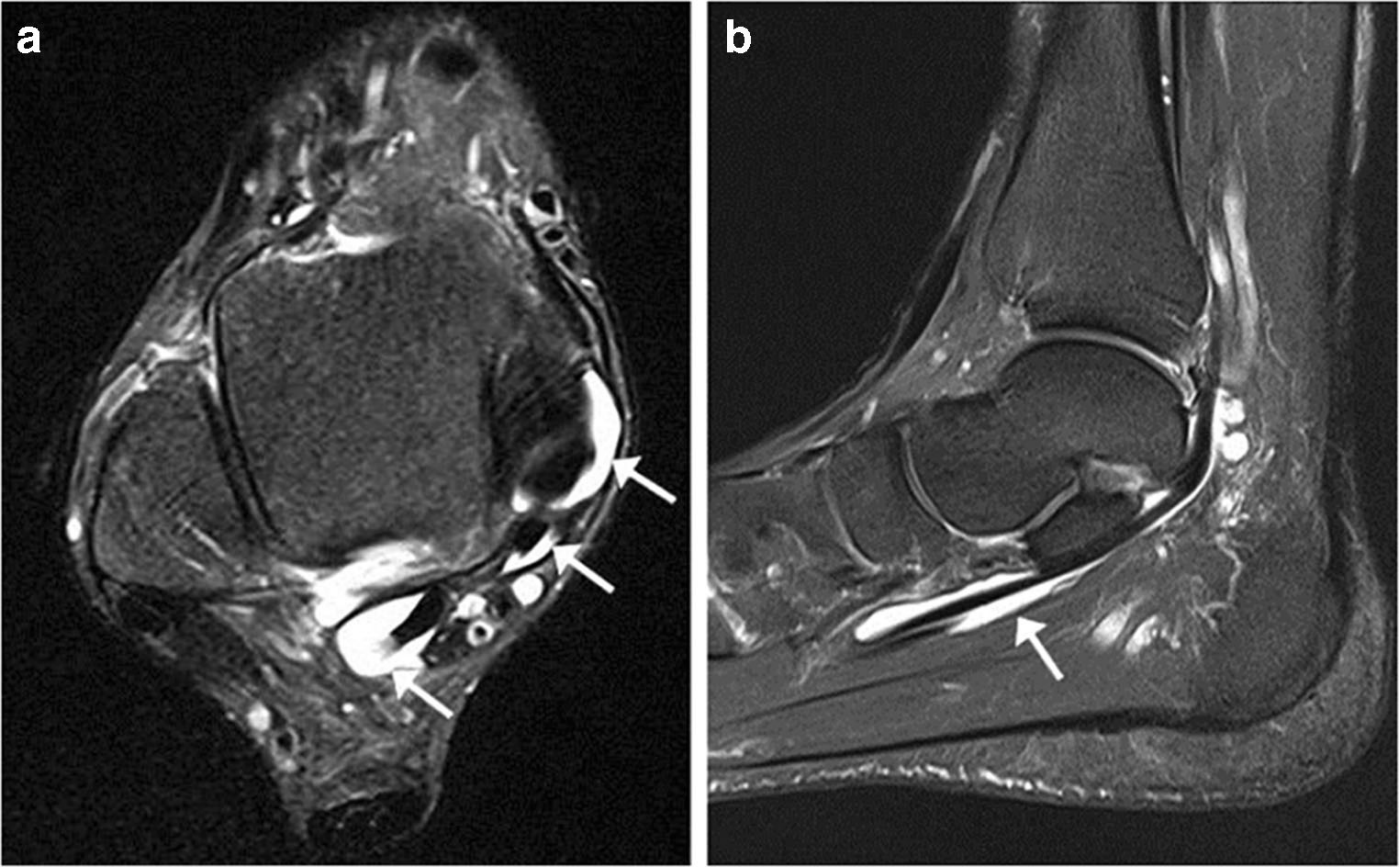
A 42-year-old male amateur marathon runner with 5 years of running experience, a running pace of 5:20 min/s/km, a weekly running distance of 65 km, and a non-rearfoot strike pattern. **A** Axial fat-suppressed T_2_-weighted imaging (fs-T_2_WI) showing fluid in the tendon sheath of the posterior tibialis tendon (grade 1), flexor digitorum longus tendon (grade 0), and flexor hallucis longus tendon (grade 2) (from the inside to the outside; arrow). **B** Sagittal fs-PDWI showing effusion in the flexor hallucis longus tendon sheath (grade 2; arrow)

**Fig. 3:**
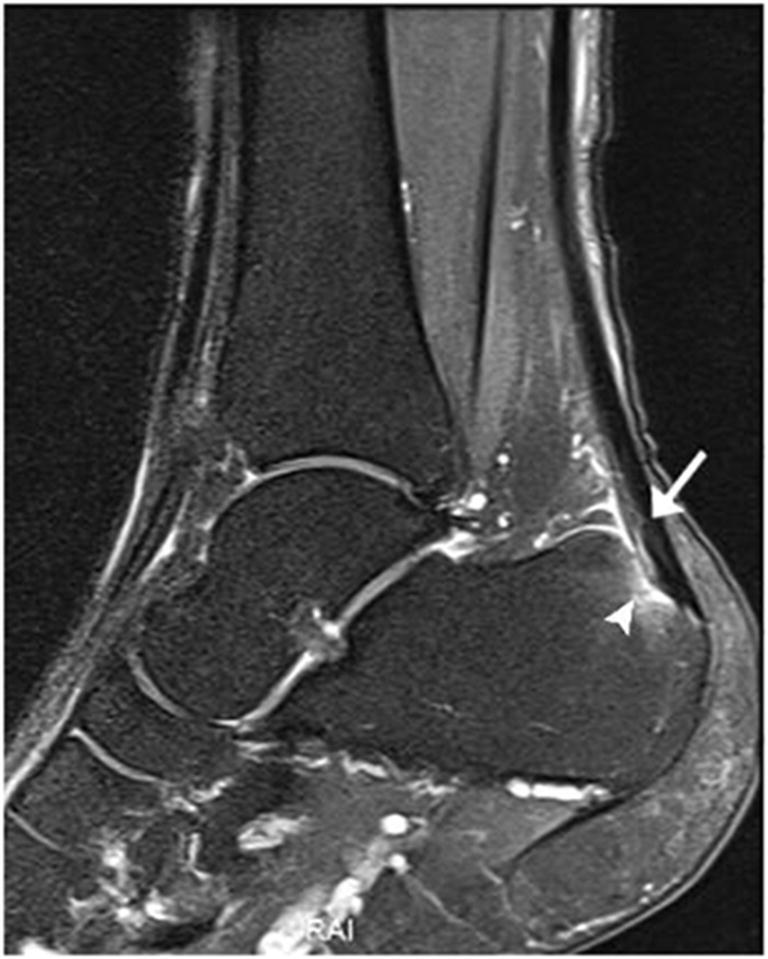
A 48-year-old female amateur marathon runner with 6 years of running experience, a running pace of 6:30 min/s/km, a weekly running distance of 40 km, and a non-rearfoot strike pattern. Sagittal fs-PDWI showing increased signal in the Achilles tendon insertion (arrow) and bone marrow edema-like signal intensity in the adjacent calcaneus (arrowhead)

**Fig. 4:**
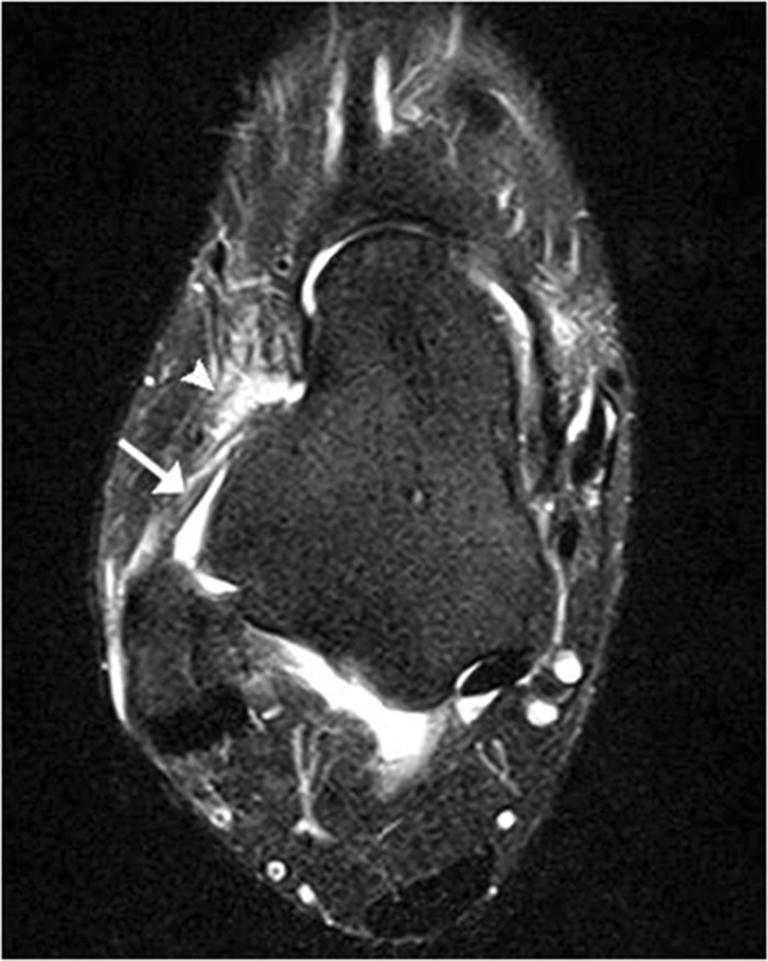
A 47-year-old female amateur marathon runner with 9 years of running experience, a running pace of 6:40 min/s/km, a weekly running distance of 50 km, and a rearfoot strike pattern. Axial fs-T_2_WI showing continuous walking and an increased signal in the anterior talofibular ligament (arrow), and an edema-like signal in the surrounding soft tissue (arrowhead) (grade 1)

**Fig. 5:**
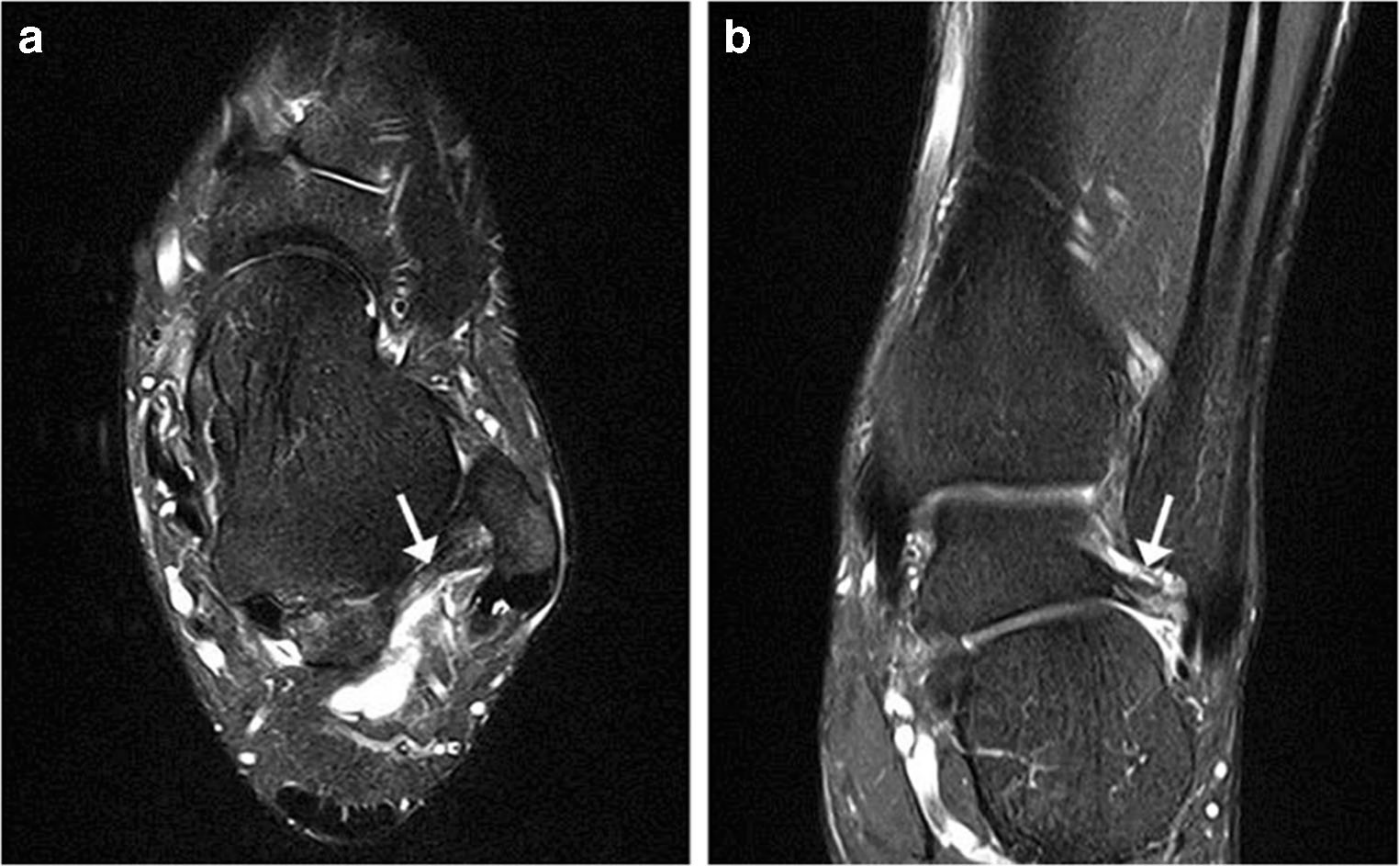
A 45-year-old male amateur marathon runner with 4 years of running experience, a running pace of 5:30 min/s/km, a weekly running distance of 60 km, and a non-rearfoot strike pattern. Axial fs-T_2_WI (**A**) and coronal fs-PDWI (**B**) showing incomplete disruption and an increased signal in the posterior talofibular ligament (grade 2; arrow)

**Fig. 6:**
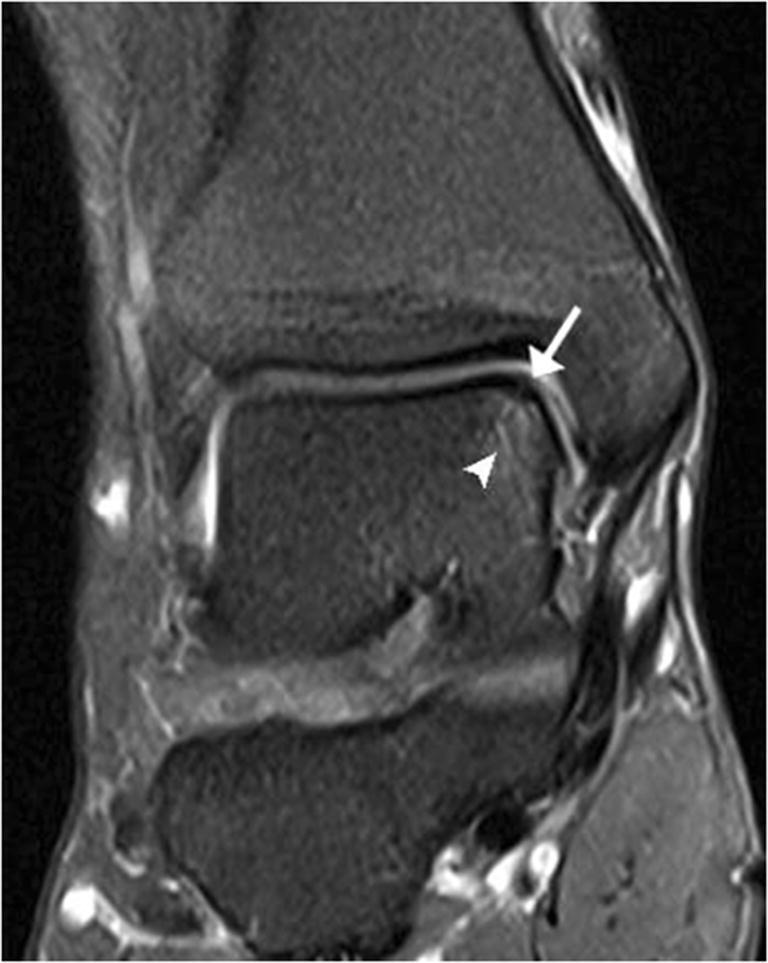
A 42-year-old female amateur marathon runner with 3 years of running experience, a running pace of 6:00 min/s/km, a weekly running distance of 40 km, and a rearfoot strike pattern. Coronal fs-PDWI showing focal thickening of the subchondral bone (arrow) and adjacent bone marrow edema-like signal intensity in the talus (arrowhead)

Bone injuries were mainly bone marrow edema-like signal intensity and cyst-like lesions. The bone marrow edema-like signal intensity (Fig. 1) was mainly grade 1 or 2 and more common in the calcaneus (35/183, 19.1%), talus (29/183, 15.8%), and distal tibia (12/183, 6.6%). There were 10 ankles with cyst-like lesions. Tendon changes were mainly peritendinous effusion. The peritendinous effusion was mainly found in flexor tendons (Fig. 2) and was mainly grade 1: posterior tibialis tendon (145/183, 79.2%), flexor hallucis longus tendon (131/183, 71.6%), and flexor digitorum longus tendon (78/183, 42.6%). There were 32 ankles with peroneus longus and brevis tendon sheath effusion. Grade 0 peritendinous effusion was mainly found in the extensor tendons, particularly the anterior tibialis tendon, extensor hallucis longus tendon, and extensor digitorum longus tendon. Achilles tendon changes were mainly manifested as Achilles tendinopathy and Achilles tendon effusion. Forty-nine ankles had Achilles tendinopathy (Fig. 3), and 21 ankles had Achilles tendon effusion. Ligament injuries mainly manifested as partial lateral collateral ligament injuries and were mainly grade 1 or 2: anterior talofibular ligament (87/183, 47.5%; Fig. 4), calcaneofibular ligament (72/183, 39.3%), and posterior talofibular ligament (46/183, 25.1%; Fig. 5). Twenty-eight ankles had deltoid ligament injuries. Other injuries included osteochondral lesions (Fig. 6) in 6 ankles and subcutaneous soft tissue edema in 25 ankles.

A marathon runner’s foot strike pattern is a potential factor that could cause ankle injury [6, 7]. While most marathon runners make initial ground contact with their heel (i.e., a rearfoot strike pattern), there are also marathon runners that make initial contact with their forefoot or midfoot (i.e., a non-rearfoot strike pattern) [8]. In this study, marathon runners who habitually used a rearfoot strike pattern were at higher risk for bone marrow edema-like signal intensity (*p* = 0.028, OR = 1.172), anterior talofibular ligament injury (*p* = 0.017, OR = 1.346), and calcaneofibular ligament injury (*p* = 0.035, OR = 1.463) than marathon runners who habitually used a non-rearfoot strike pattern. However, rearfoot strike marathon runners had a lower risk of peritendinous effusion than marathon runners that used a non-rearfoot strike pattern (*p* = 0.005, OR = 0.831). Previous studies provided evidence that a weekly running distance ≥ 60 km increased the risk of RRI [9, 10]. Therefore, the weekly running distance was divided into a higher weekly running distance group and a lower weekly running distance group at 60 km. Our study indicates that marathon runners with higher weekly running distances are at higher risk for peritendinous effusion (*p* = 0.013, OR = 1.685), anterior talofibular ligament injury (*p* = 0.022, OR = 1.171), and calcaneofibular ligament injury (*p* = 0.029, OR = 1.570) than marathon runners with lower weekly running distances. The study also showed that the risk of peritendinous effusion increases with increased running years (*p* = 0.039, OR = 1.113) and that the risk of Achilles tendinopathy increases with age (*p* = 0.008, OR = 1.412). Sex and running pace both had *p >* 0.05, which was not statistically significant (Table 4).

**Table 4:** Summary of related factor analysis results

MRI features and related factors	*p*	OR	95% CI
Bone marrow edema-like signal intensity			
Sex	0.869	1.065	0.505–2.245
Increased age	0.105	1.046	0.991–1.104
Increased running years	0.336	0.882	0.682–1.140
Slower running pace	0.997	1.002	0.437–2.298
Higher weekly running distance	0.521	1.307	0.577–2.965
Rearfoot strike pattern	*0.028*	1.172	1.012–1.731
Peritendinous effusion			
Sex	0.274	0.658	0.311–1.392
Increased age	0.783	1.010	0.942–1.083
Increased running years	*0.039*	1.113	1.021–1.566
Slower running pace	0.586	0.794	0.347–1.820
Higher weekly running distance	*0.013*	1.685	1.448–2.182
Rearfoot strike pattern	*0.005*	0.831	0.533–0.968
Achilles tendinopathy			
Sex	0.214	1.658	0.747–3.681
Increased age	*0.008*	1.412	1.143–1.985
Increased running years	0.846	0.973	0.740–1.208
Slower running pace	0.160	1.853	0.783–4.382
Higher weekly running distance	0.214	0.587	0.253–1.360
Rearfoot strike pattern	0.118	1.913	0.848–4.316
Anterior talofibular ligament injury			
Sex	0.293	0.670	0.318–1.414
Increased age	0.883	0.996	0.949–1.046
Increased running years	0.344	1.132	0.875–1.465
Slower running pace	0.321	0.655	0.284–1.509
Higher weekly running distance	*0.022*	1.171	1.066–1.510
Rearfoot strike pattern	*0.017*	1.346	1.134–1.805
Calcaneofibular ligament injury			
Sex	0.072	1.810	0.552–4.098
Increased age	0.066	1.009	0.835–1.102
Increased running years	0.486	1.096	0.847–1.419
Slower running pace	0.467	1.367	0.589–3.177
Higher weekly running distance	*0.029*	1.570	1.250–2.431
Rearfoot strike pattern	*0.035*	1.463	1.197–2.204

Italicized data are presented as *p* < 0.05, which was statistically significant

## Discussion

The subjects of this study were all asymptomatic amateur marathon runners without any clinical symptoms. However, many of the subjects showed changes in bone marrow edema-like signal intensity, peritendinous effusion, and partial lateral collateral ligament injury. In addition, the factors that caused injuries to ankle joints varied and included sex, training distance, training frequency and duration, running pace, foot strike pattern, road conditions, types of running shoes, and previous injury [11, 12].

The bone marrow edema-like signal intensity of amateur marathon runners was more common in the distal tibia, talus, and calcaneus, mainly grade 1 or 2 and usually asymptomatic. At present, the mechanism of bone marrow edema-like signal intensity is unknown. Bone marrow, like other soft tissues such as muscle, needs to be metabolized and can change dynamically as the internal environment changes. There is a theory that bone marrow edema-like signal intensity is caused by certain external forces acting on the bone to cause microfracture of bone trabecula, increased capillary permeability of the bone marrow, extracellular fluid leakage, and increased local bone marrow tissue perfusion and can be accompanied by capillary rupture and bleeding. Another point of view is physiologically reactive bone marrow edema-like signal intensity. Long-term external force effects, such as long-term external pressure and long-term changes in bone load, can cause the corresponding bone marrow tissue to produce a certain physiological response. This kind of bone marrow edema-like signal intensity is a reversible change, and often disappears after avoiding external force or loading [13–15]. Bone marrow edema-like signal intensity can be seen in recreational athletes 1–8 weeks after running [16, 17]. Previous studies showed that any remaining bone marrow edema-like signal intensity of the knee appearing post-marathon was expected to resolve within 2 years [18–21]. Our study shows that rearfoot strike marathon runners have a higher risk of bone marrow edema-like signal intensity than non-rearfoot strike marathon runners. There is some support for this conclusion, as in marathon runners, a non-rearfoot strike pattern appears to result in a lower ground reaction force than a rearfoot strike pattern [22, 23]. Moreover, marathon runners who habitually use a rearfoot strike pattern appear to demonstrate a greater incidence of repetitive pressure injury than marathon runners who habitually use a non-rearfoot strike pattern [6]. Therefore, marathon runners who habitually use a rearfoot strike pattern are more prone to bone marrow edema-like signal intensity due to increased ground reaction force and repetitive pressure on the ankle bone. No stress fracture occurred in any subjects in this study nor was there any correlation between bone marrow edema-like signal intensity and the weekly running distance, which may be related to the small sample size. However, a previous report indicated that runners with a weekly running distance of more than 40 km had an increased risk of stress fracture [24]. According to this theory, asymptomatic bone marrow edema-like signal intensity can evolve, with continuous overuse, to a symptomatic stress reaction or stress fracture [14].

Amateur marathon runners had a high incidence of peritendinous effusion in our study. It was mainly found in flexor tendons and mainly grade 1, and it may be related to overuse caused by repeated pulling of the flexor tendons. The posterior tibialis tendon, flexor hallucis longus tendon, and flexor digitorum longus tendon all function in plantar flexion of the ankle. When using a non-rearfoot strike pattern, the ankle is flexed as soon as it touches the ground. In this process, the flexor tendon may be pulled repeatedly, causing overuse, and resulting in an increase in the corresponding fluid around the tendon sheath. The results of this study also indicate that the risk of peritendinous effusion is related to the weekly running distance and running years, which may be due to the increased risk of overuse injury in amateur marathon runners with longer running distances or more running years and not enough time for repair. The next step for research is to further investigate whether the peritendinous effusion of amateur marathon runners is a reversible change.

Achilles tendinopathy is one of the most common diseases reported by runners, accounting for approximately 10% of running injuries [25]. Achilles tendinopathy may result from degeneration secondary to excessive and repetitive loading without adequate repair [26, 27]. Our results appear to show that the risk of Achilles tendinopathy only increases with age. Longo et al. [28] reported that there was no evidence of a statistically significant association between sex, weight, height, number of marathons, and Achilles tendinopathy in 350 marathon runners participating in the 2017 Roman Marathon. However, age was associated with Achilles tendinopathy, which is consistent with the results of our study. One would expect that Achilles tendon lesions in amateur marathon runners are only a degenerative change with age rather than a traumatic change, and they do not show the corresponding clinical symptoms. A previous study indicated that asymptomatic male long-distance runners had a high incidence of tendinopathy, and increased running years was associated with pathology in the Achilles tendon [29]. A study by Kernozek et al. [7] showed that female runners who habitually use a non-rearfoot strike pattern may be at greater risk for Achilles tendinopathy than runners who habitually use a rearfoot strike pattern and differences in Achilles tendon loading between non-rearfoot and rearfoot strike runners may be a contributing factor. However, our results indicate that there was no evidence of a statistically significant association between running years, foot strike pattern, and Achilles tendinopathy in asymptomatic amateur marathon runners. This result may be due to the small sample size we had and needs to be further verified by increasing the sample size later.

The incidence of ligament injury in our study was relatively high, and these injuries were mainly grade 1 or 2 and manifested as partial lateral collateral ligament injuries. In our results, there is evidence of a statistical association among higher weekly running distance, rearfoot strike pattern, and an increase in the risk of partial lateral collateral ligament injury. In a marathon, runners are subjected to a ground reaction force that is approximately two to three times their own weight, and the impact of the ground reaction forces is repeated throughout the race [23]. When running with a rearfoot strike pattern, the ankle joint directly bears the increased reaction force from the ground, and marathon runners who use a rearfoot strike pattern or have a higher weekly running distance will also have an increased risk of ligament injury due to overuse. Therefore, long-term, repeated training and competition easily lead to ligament injury.

A small number of amateur marathon runners had cyst-like lesions, osteochondral lesions of the talar dome, subcutaneous soft tissue edema around the ankle joint, and so on. Due to the small number of effective samples, no meaningful statistical conclusions have been drawn. Cyst-like lesions are usually related to joint degeneration [30]. Osteochondral lesions of the talar dome may be associated with repeated articular cartilage surface loading or excessive stress [31, 32]. Subcutaneous soft tissue edema around the ankle joint may be related to water intake and running distance [33]. Furthermore, this study did not find a correlation among running pace, sex, and ankle injuries, which may be because the running pace of all subjects was concentrated in a relatively small range with little difference. A study by Schwabe et al. [34] showed that running at either a slow or fast pace was a risk factor for complications during 56-km ultramarathon running. The prospective cohort study by Buist et al. [35] found a moderately significant increased risk of general injury among males compared with females. In contrast, Lopes et al. [36] thought that the presence of pain was significantly greater in female recreational runners than males. However, a systematic review by Hulme et al. [12] did not find statistically significant differences in the RRI risk across running pace and sex specifically.

Our study has limitations. First, a symptomatic group and a normal control group were not included. Second, only a few related factors that affected ankle injuries were included in this study. Other possible factors, such as BMI, number of marathons, running experience, and previous injury, were not included in the analysis. In addition, the ankles of amateur marathon runners before and after the marathon running were not compared, and dynamic tracking of multiple injuries was not carried out, so the evolution of ankle joint injuries was not studied. Finally, the MR diagnosis of ankle injuries was a qualitative observation, without a quantitative analysis, and had a certain subjectivity. All of these issues need to be addressed, and further studies are needed in the future.

In conclusion, marathon running is a sport that not only challenges human physical and physiological limits, tempers the will and tests endurance but also may be associated with risk if done improperly. Amateur marathon runners may be prone to ankle injuries in daily training and competition without any clinical symptoms. The main MRI features of related ankle injuries include bone marrow edema-like signal intensity, peritendinous effusion, and partial lateral collateral ligament injury. In addition, increased age, increased running years, higher weekly running distance, and different foot strike patterns are risk factors for ankle injuries.
